# The Vaginal Route in Pelvic Surgery

**Published:** 1904-10

**Authors:** O. L. Norsworthy

**Affiliations:** Houston, Texas


For Texas Medical Journal.

### The Vaginal Route in Pelvic Surgery.

O. L. NORSWORTHY, M. D., HOUSTON, TEXAS.

I offer no claim of priority to this manner of operating, nor do I claim the results of my work in this field to be better than yours done by the abdominal route; though I do believe the results following my operating through the vagina better than through my abdominal incisions in many cases. I shall not attempt to discuss in this paper the pros and cons connected with the two fields. Nor shall I attempt to cover the field of pelvic surgery done through the vagina. I have confined my remarks to just such cases as have come under my personal observation, and have reported only cases operated on by myself. I will mention three of the most important reasons for using the vaginal incision in preference to the abdominal, according to my conclusions drawn from four years' limited work in the field. 1. The dangers to the patient's life are greatly lessened. 2. If the case is a suitable one, the cure can be made just as radical. 3. There is no external visible cicatrix to be constantly reminding the patient of her operation. Pelvic work done through the vagina alone can not be applied successfully to any and all conditions, but it can be in many, and it will greatly facilitate the work, and often lessen the danger by being used in addition to the abdominal opening in many cases.

Before reporting my cases, I will make a few general remarks regarding some points I have found useful while performing this operation. While doing so I only give you my personal experience and ideas, as I have not gathered any text-book descriptions nor journal reference on the subject. I prepare patient's abdomen and vagina by shaving the entire surroundings from umbilicus to anus, and using ordinary asepsis into the vagina. Empty bowels well. Always anesthetize before fully deciding the route for operating. Dilate cervix, curette and irrigate uterus. I have found a self-retained speculum, sharp and blunt pointed scissors, tenaculum forceps, ovarian forceps, long vaginal retractors and clamps very necessary aids to the work. Make strong traction on cervix while you open through recto vaginal septum into Douglas pouch with scissors and fingers, clinging closely to cervical tissue. With fingers locate fundus of uterus, trace tubes out, break up adhesions and locate ovaries. If necessary, bring ovaries and tubes into field

---

*Read before the South Texas Medical Association, July, 1904.

of eye to decide about removing them. If the operator is right-handed, remove left adnexa first by clamping uterine end first, cut, clamp fimbriated end and remove. If necessary, include ovary in last clamp and remove it. If drainage will permit, place a catgut stitch in either angle of incision. Pack gauze into opening, wrap clamps well with gauze and tie handles together. Remove clamps from twelve to twenty-four hours and gauze from three to five days, or as the drainage may indicate.

To economize time, I will use many times the term "uneventful recovery," meaning in these particular cases: No hemorrhage nor intense pain. No high temperature nor rapid pulse. No abdominal distension nor any of the symptoms so often following prolonged manipulation through an abdominal incision.

In gathering cases from my notes, I have selected such ones as will enable me to show you some pathological formations which can be removed successfully and a radical cure effected, without opening the abdomen. I have included in the report of some of my cases a rather incomplete history that you may judge whether or no I was justifiable in performing any operation, though the majority of cases I have reported very briefly on account of your valuable time.

Case 1.—Mrs. C., white, age 33, four children and no abortions. Mother died of tuberculosis. Had been curetted twice and perineum repaired before I saw her, which lessened her symptoms but little. At times I could find left tube large and fluctuating, though not always so. Local and general treatment for several months gave no relief. Tubercular pyo salpinx diagnosed beyond a reasonable doubt. In June, 1900, I dilated and curetted uterus and opened Douglas pouch. No adhesions, enlarged tubes nor abnormal ovaries were found. I clamped and removed left tube and ovary, believing it to be tubercular and emptying itself through uterus. Catgut stitch in either angle of incision and placed gauze loosely in opening. Clamps removed in ten hours; third day gauze removed, and fifth day sitting up in bed. An uneventful recovery with only a few months relief of symptoms followed.

Case 2.—Mrs. L., white, age 28, three children and two abortions. Dysmenorrhea and almost constant pain in region of left ovary since last abortion. Leucorrhea. Left ovary prolapsed, enlarged and painful to pressure. Six months local treatment did no good. I dilated and curetted uterus and opened into the pouch of Douglas in October, 1900. Left ovary was prolapsed, enlarged with dark venous blood and cysts; right somewhat enlarged. Left ovary only removed after clamping. Catgut stitch was inserted in either angle. Clamps removed in twelve hours, gauze third day; opening closed on sixth day and patient wanting to sit up. An uneventful recovery, and a cure was the result.

CASE 3. Same patient as case 1. Diagnosis, tubercular right tube. In April, 1901, I again dilated and curetted uterus and reopened pouch. No adhesions. Clamped and removed right ovary and tube. Two catgut stitches were used and gauze inserted. Clamps removed in twelve hours; gauze on third day; fifth day opening closed and patient wanting to sit up. An uneventful recovery, and a change of climate to San Antonio followed. Patient now is greatly improved, though not entirely well.

CASE 4.—Same patient as case 2. Following another abortion, dysmenorrhea and ovarian pain in right side were very marked. Five months' treatment did not good. In September, 1901, I dilated and curetted uterus and reopened Douglas pouch. I found right ovary prolapsed and almost completely destroyed by a hematoma. I clamped, removed ovary with tumor and used stitches. Clamps removed in twelve hours; gauze on third day; opening closed on the sixth day and patient ready to get up. An uneventful recovery followed. Patient is now well; better health than she has been since marriage.

CASE 5.—Mrs. L., white, age 36, one child and three abortions. Has suffered with backaches and dysmenorrhea for two years. Refused a laparotomy for a tumor. In November, 1901, I could discover a large immovable mass behind the uterus. I curetted uterus and dissected up to mass and into a sac of serum. To further assure myself I contined dissection into peritoneal cavity. Ovaries and tubes released from few adhesions and gauze placed in opening until closed. An uneventful recovery, and a complete cure followed.

CASE 6.—H., negress, age 33, diagnosis many small myomata on posterior wall of uterus. In June, 1901, I opened through the vagina and soon realized an abdominal incision was necessary. I dissected out seven small myomata, through the abdomen, stitched up their beds and left uterus, ovaries and tubes intact. Patient died on fifth day of actute general peritonitis. I learned afterwards that no asepsis was observed by one of the nurses assisting in the operation. I believe with my present experience I could have dissected out all of these tumors through a vaginal opening and most likely saved my patient's life.

CASE 7.—Mrs. B., white, age 48, two children. Mother died of cancer of uterus. Backaches and profuse menstrual flow. I

curetted, repaired cervix and perineum successfully in August, 1901, with no effect of symptoms. During the winter of 1902 uterine hemorrhage was continuous. She was curetted and packed several times by her physician, which gave her no relief. I was called to remove uterus; I discouraged the idea, believing patient would die on the table. Patient and attending physician insisted that it be done, as she was bleeding to death as she was. Vagina was split and uterus removed through it. Hypodermics of stimulants being used freely to sustain life while on the table. Clamps removed on following morning; packing continued until opening healed up. An uneventful recovery, though considerable sloughing and suppuration followed. There are no evidence of cancer at this present writing.

CASE 8.—Mrs. B., white, age 34, two children and no abortions. In October, 1902, I removed a follicular growth from anterior cervical lip, repaired cervix and perineum successfully, hoping to increase the menstrual flow, thereby relieving cataleptic spells, to which this patient was subject. No improvement followed. In January, 1903, I curetted again. No improvement followed. In April, 1903, I opened Douglass pouch and spent several minutes removing cysts from ovaries. Finally decided there was no normal tissue left; I clamped and removed ovaries. Following morning patient had a convulsion and jerked one clamp off as I was about to remove it. Alarming hemorrhage followed. Opening was packed high up and vagina left empty. All preparations made for a laparotomy. Hemorrhage ceased and patient recovered without an abdominal opening. Though no indications of shock until the hemorrhage, which was twenty-nine hours after operation, all signs of shock following hemorrhage then were in evidence, and marked abdominal distension continued for three days. Recovery followed; also the cataleptic convulsions continue. Patient's general health has been much improved.

CASE 9.—Mrs. H., white, age 24. No children but two abortions. Diagnosis, severe right ovarian neuralgia. No relief from a year's treatment by her physician. In February, 1903, I curetted uterus and opened into recto-vaginal pouch; found right ovary prolapsed, enlarged, very dark and slightly adherent. I loosened adhesions, removed two small cysts from it, pushed it up and braced it well with gauze. Removed gauze on seventh day. Replaced gauze and removed it on fifth day. Recovery followed. Though for several months following there was some pain in the right side, the patient has recovered entirely and does not suffer now.

CASE 10.—Mrs. H., white. One child. Right ovarian neuralgia. Seven months' treatment by her physician gave no relief. I curetted uterus, opened posterior pouch, clamped and removed a very large diseased ovary—in February, 1903. Clamps removed following morning; gauze on third day; opening had closed up by sixth day and patient ready to get up. An uneventful recovery followed.

CASE 11.—Mrs. M., white, age 42, one child and two abortions. Diagnosis, pus tube complicated with profuse and almost continuous uterine hemorrhage. Patient had been hemorrhaging for ten weeks continuously, and was very weak and anemic. Uterus curetted and pouch opened in May, 1903; adhesions loosened until I could outline pus tube. On account of its being attached extremely high, to a level of crest of ilium, I was unable to remove it. Patient too weak and anemic to attempt a laparotomy, I opened and packed. Thinking I was opening into second pus sac, I punctured the rectum. Packing continued until healing, from bottom, was thought to have taken place. Patient made an uneventful recovery, and is now in best of health, except uterine hemorrhages occasionally.

CASE 12. Mrs. K., white, age 26, one child; no abortion. Diagnosis, pus tube. I was asked to remove it in October, 1903. While dissecting through recto-vaginal septum, I cut into a split leading into uterine cavity. I continued posteriorily to uterus into pelvic cavity, and found no pus tube. A mass of adhesions was broken down, uterine split sutured up and opening packed well. An uneventful recovery followed and patient cured, except a slight leucorrhea continues.

CASE 13.—Mrs. F., white, age 35, two children; no abortions. Had pelvic abscess opened two years ago. Dysmenorrhea accompanied by hysterical convulsions were present every month. In March, 1904, I curetted and repaired a badly lacerated cervix and loosened up all adhesions through the vagina. The left ovarian ligament being too short to permit the placing of ovary in new bed, I clamped and removed it from a bed of dense, firm adhesions. Right adnexa normal. Clamps removed following morning and gauze on fifth day. Patient made an uneventful recovery. She is not entirely free from pain as yet though it is growing less every month.

CASE 14.—Mrs. I., white, age 26, one child and no abortions. Gives a history of gonorrhea. A pelvic abscess was opened through vagina two years ago. In May, 1904, I dissected much diseased ovary, being unable to place ovary in a new bed. Right tube was hardened but not enlarged; right ovary not located. Clamps removed on following morning and gauze on fourth day. Patient ran a temperature from 99 to 102 for several days, but left the hospital on the eighteenth day, She is now around and doing well; without pelvic pain. August 15th. Patient is entirely well, and claims she is feeling better than for several years.

Case 15.—Mrs. C., white, age 35, one child and no abortion. Diagnosis, myomatous uterus, projecting posteriorly and anteriorly to the left to the height of the anterior superior spine of ilium, of thirteen years' standing. On July 5, 1904, I bisected and removed uterus and peeled out in morsels the tumor, consisting of considerably more than a half gallon measure, leaving the ovaries, tubes and entire peritoneum intact, excluding a small portion clinging to posterior wall of uterus and an occasional perforation of it in other places. The difficulties encountered in the operation were:

First. Hemorrhage from an abnormally large and high division of the left ovarian artery, which occurred after tying the main artery. Second. Loosening, without injury, the left ureter from the high projecting tumor which was adhered to and partially imbedded in the tumor. Neither of the above complications could have been prevented nor quicker corrected through an abdominal opening than the vaginal. The patient was considerably shocked. She soon rallied and made a complete and uneventful recovery.

